# Naringin provides neuroprotection in CCL2-induced cognition impairment by attenuating neuronal apoptosis in the hippocampus

**DOI:** 10.1186/s12993-020-00166-6

**Published:** 2020-02-27

**Authors:** Jiang-yi Long, Jian-min Chen, Yuan-jun Liao, Yi-jun Zhou, Bing-yu Liang, Yan Zhou

**Affiliations:** 1 Department of Pharmacology, Guangxi Medical University, Nanning, 53002 Guangxi China; 2 Guangxi Key Laboratory of AIDS Prevention and Treatment, Guangxi Medical University, Nanning, 530021 Guang China

**Keywords:** Naringin, Cognitive impairment, Anti-inflammation, Antioxidants, Apoptosis, Glutamate metabolism

## Abstract

**Background:**

Chemokine C–C motif ligand 2 (CCL2) is one of the most widely recognised proinflammatory chemokines in cognitive disorders. Currently, CCL2-targeting drugs are extremely limited. Thus, this study aimed to explore the neuroprotection afforded by naringin in CCL2-induced cognitive impairment in rats.

**Methods:**

Before the CCL2 intra-hippocampal injection, rats were treated with naringin for 3 consecutive days via intraperitoneal injection. Two days post-surgery, the Morris water maze (MWM) and novel object recognition (NORT) tests were performed to detect spatial learning and memory and object cognition, respectively. Nissl staining and dUTP nick-end labelling (TUNEL) staining were performed to assess histopathological changes in the hippocampus. Commercial kits were used to measure the activity of superoxide dismutase (SOD) and glutathione peroxidase (GSH-Px) and the content of malondialdehyde (MDA). Quantitative real-time polymerase chain reaction (qRT-PCR) was performed to examine the relative mRNA expression of interleukin 1β, (IL-1β), interleukin 6 (IL-6), glutamate/aspartate transporter (GLAST), glutamate transporter-1 (GLT-1), phosphate-activated glutaminase (PAG), cysteine aspartic acid-specific protease 8 (caspase-8), cysteine aspartic acid-specific protease 3 (caspase-3), cell lymphoma/leukaemia-2 (Bcl-2), and Bcl-2 associated X protein (Bax).

**Results:**

In the MWM, the average escape latency and average swimming distance were significantly reduced and the crossing times were increased in the naringin-treated groups, compared with the CCL2 group. The NORT results revealed that, compared with the CCL2 rats, the discrimination index in the naringin-treated rats increased significantly. Nissl and TUNEL staining revealed that naringin protected the structure and survival of the neurons in the CA1 zone of the hippocampus. In the naringin-treated groups, the SOD and GSH-Px activities were increased, whereas the MDA levels were decreased. Furthermore, in the naringin-treated groups, the relative mRNA expression of IL-1β and IL-6 was significantly decreased; GLAST and GLT-1 mRNA expression levels were increased, whereas PAG was decreased. In the naringin-treated groups, the relative mRNA expression levels of caspase-8, caspase-3, and Bax were decreased, whereas that of Bcl-2 was increased.

**Conclusion:**

Collectively, these data indicated that naringin alleviated the CCL2-induced cognitive impairment. The underlying mechanisms could be associated with the inhibition of neuroinflammation, oxidative stress, apoptosis, and the regulation of glutamate metabolism.

## Background

The chemokine C–C motif ligand 2 (CCL2), also known as the monocyte chemoattractant protein-1(MCP-1) [1, 2], belongs to the C–C chemokine family [3]. It has the potent ability to activate and recruit mononuclear phagocytes and activate T cells and B cells; functions that are well-characterised in the immune system [4, 5]. However, recent studies have revealed that CCL2 is also involved in several central nervous system (CNS) diseases, such as epilepsy, Alzheimer’s disease, and ischaemic brain injury [1]. Elevated CCL2 has been detected in the cerebrospinal fluid (CSF) of patients with Alzheimer’s disease (AD) and HIV-associated neurocognitive disorder (HAND) [6–9]. In the brain, CCL2 is produced mainly by macrophages and microglia, which, in turn, activate microglia and release numerous inflammatory cytokines [10, 11], such as interleukin 6 (IL-6) and interleukin 1β (IL-1β), exacerbating the extent of inflammation and resulting in neuronal injury. Thus, as a potential proinflammatory mediator, we postulated that CCL2 is closely associated with neuroinflammation and cognitive impairment.

In addition to neuroinflammation, our previous study has indicated a novel role of CCL2 in mediating excitotoxicity in an in vitro study. The administration of CCL2 enhances *N*-methyl-d-aspartic acid (NMDA) receptor-mediated excitatory postsynaptic currents (EPSCs) and ultimately impairs neuronal dendrites in the hippocampal CA1 region, inducing neuronal death in the hippocampus [12]. Based on these findings, we hypothesised that CCL2 impairs neurons via multiple pathways and could be a potential therapeutic target in several CNS diseases.

However, drugs currently available against CCL2 are extremely limited. Naringin, a flavonoid naturally existing in grapefruit and other citrus fruits, possesses numerous biological benefits. Preclinical evidence has suggested the protective role of naringin in the prevention of cardiovascular disease, diabetes, and neurodegeneration via antioxidant and anti-inflammation properties [13–17]. Another study has demonstrated that naringin can alleviate the progression of atherosclerosis by downregulating CCL2 expression [18]. However, the potential protective effects of naringin against CCL2-induced neuronal impairment have not been investigated. Therefore, in this study, we investigated the effect of naringin on CCL2-induced cognitive impairment and elucidated the possible underlying mechanisms.

## Materials and methods

### Reagents and instruments

CCL2 (R&D system) and naringin (purity > 98%) were purchased from Sigma Chemicals (USA). RT-PCR primers were obtained from Generay Biotech (Japan). The TRIzol reagent for RT-PCR was purchased from TaKaRa Bio Inc (Japan). The commercial kits were purchased from Nanjing Jiancheng Bio-engineering Institute (Nanjing, China). TUNEL and the Nissl kit were procured from Beyotime Institute of Biotechnology (Haimen, China).

### Experimental animal grouping

**Fig. 1:**
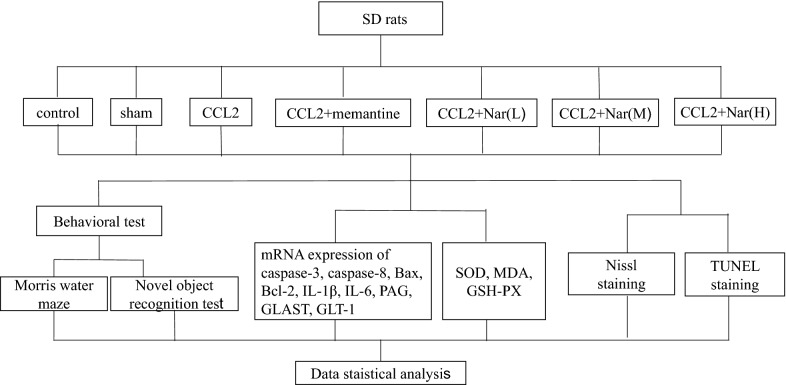
Experimental designs

All animal experiments were performed in accordance with the guidelines of the Animal Ethical Committee of Guangxi University. Seventy-seven SPF male Sprague–Dawley rats (4–6 weeks old, weighing 180–220 g) were provided by the Guangxi Medical University. The rats were maintained in an air-conditioned room (22 ± 2 °C, 12-h light/dark cycle) with free access to water. The rats were randomly divided into seven groups (n = 11), including the control group, sham group, model group (5 ng CCL2), positive drug treatment group (CCL2 + 10 mg/kg memantine), naringin low dose group (CCL2 + 25 mg/kg naringin), naringin middle dose group (CCL2 + 50 mg/kg naringin), and naringin high dose group (CCL2 + 100 mg/kg naringin), as shown in Fig. 1.

### Stereotaxic surgery and drugs treatment

Firstly, CCL2 was dissolved in 100 ng μL^−1^ using sterile saline and then diluted to 1 ng μL^−1^ before the experiment. With the exception of the control group, each group underwent a bilateral hippocampal injection. Briefly, rats were anaesthetised using an intraperitoneal (i.p.) injection of 1% sodium pentobarbital (45 mg kg^−1^). The fur on the rat’s head was removed following anaesthesia. According to the stereotaxic map of rat brain, a 26 GS micro-syringe was used to inject the test drugs into the hippocampus following the coordinate positions of AP = − 3.7 mm, ML = ± 3.0 mm, and DV = − 3.0 mm. The injection volume was 2.5 μL per side, with a constant speed of 0.3 μL/min; the sham group received an equal volume of sterile saline. After administration, the needle was left in place for another 5 min to avoid leakage of the drug and ensure complete absorption. Next, we sutured the skin and administered penicillin (300,000 units kg^−1^, i.p.) to prevent the development of any infection. Before the administration of CCL2, the rats in the treatment groups were treated with naringin and memantine repeatedly for 3 consecutive days via i.p. injection. The rats in the control, sham, and model (5 ng CCL2) groups were administered equal volumes of normal saline for 3 consecutive days. On the third day, drug administration was performed 30 min before the bilateral hippocampal injection.

### Morris water maze (MWM)

In rats, spatial learning and memory assess began on the third day following hippocampal injection. The methodology was as described by Vorhees et al. [19]. The MWM paradigm consisted of a circular pool, with a diameter of 120 cm and a height of 110 cm, a video capture system, and a software analysis system. The pool was divided into four equal quadrants, including NW, SW, SE, and NE. Near the wall of each quadrant, a distinct marker of similar size and different shape was placed. The water temperature was controlled at 22 ± 1 °C. In the centre of the SW quadrant, a platform was submerged 2 cm below the water surface. The experiment consisted of three phases: (1) habituation phase: To enable environmental adaptation and avoid stress, all rats were allowed to swim in the pool for 60 s before performing the experiment; (2) spatial navigation phase: This phase was performed for five consecutive days. On each day, the rats were placed in the water from different quadrants and administered four trails each day. We recorded the time from the start to find the platform, termed the escape latency. Additionally, swimming speed and swimming distance were measured. Each trial was performed for 90 s. If the rat failed to reach the platform within 90 s, we guided the animal to the platform for 30 s and recorded the escape latency as 90 s. (3) Probe trial: This phase was performed 24 h after the end of spatial navigation. We removed the platform and introduced the rats at a random quadrant for 90 s. The crossing times to reach the position of the platform were recorded as the evaluated index.

### New object recognition test (NORT)

This experiment was conducted after the MWM for 2 consecutive days. The methods were as described by Leger et al. [20]. The apparatus consisted of a transparent plastic box (60 cm × 40 cm × 80 cm), two identical objects A, a different object B, and a video recording system. On the first day, the rat was habituated to the box for 5 min. Then, the rat was returned to the cage and the box was wiped using 75% alcohol to prevent the odour of the preceding rat to influence the next rat. Twenty-four hours later, we first placed two identical object As at the two adjacent corners of the box. The rat was given 10 min to freely explore the environment. One hour later, one object A was replaced with object B and the rat was allowed to explore for 5 min. We recorded the total time spent at A (time for the familiar; TF) and B (time for novel; TN), respectively. The discrimination index (DI) was evaluated according to the following formula: DI = TN/(TN + TF) * 100%.

### Samples preparation

Following the performance of NORT, the rats were anaesthetised with 10% chloral hydrate and decapitated. The brain was quickly removed on a culture dish filled with ice-cold saline (~ 4 °C). Three samples of the whole-brain were used for Nissl and TUNEL staining (n = 3), four samples of one side of the hippocampus were used for RT-PCR (n = 4), and eight samples of the other side of the hippocampus were used for oxidative stress detection (n = 8).

### Nissl staining and TUNEL staining

Nissl staining was conducted according to the kit protocols. Images were photographed using an Olympus BX53 fluorescence microscope at 400×. TUNEL staining was performed in accordance with the manufacturer’s protocol. An Olympus BX53 fluorescence microscope was used to capture the images under 400×. The apoptotic cells were quantitatively assessed, with three animals examined per group and three slices per hippocampal sample.

### Oxidative stress determination

To examine the effects of naringin in CCL2-induced hippocampal oxidative stress, we measured the expression levels of glutathione peroxidase (GSH‑PX), malondialdehyde (MDA), and superoxide dismutase (SOD). Briefly, the hippocampus was prepared as a 10% homogenate using iced saline. The total protein concentration of each sample was determined by the BCA method. SOD, GSH-PX activity, and MDA content were detected according to the specific kit instructions.

### qRT-PCR experiment

Briefly, total RNA extracted from the hippocampus was evaluated according to the RNA extraction kit instructions. RNA was reverse transcribed into cDNA as directed by the reverse transcription kit. The PCR reaction was quantified using the SYBE Green reagent and analysed by the StepOnePlus™ Real-Time PCR System. GAPDH was used as the reference gene. The relative mRNA expression was calculated using the 2^−ΔΔ^Ct method. Table 1 presents the primer sequence.

**Table 1:** The primer sequences of target genes

Gene	Primer sequence
GAPDH	F: 5′-GACATGCCGCCTGGAGAAAC-3′ R: 5′-AGCCCAGGATGCCCTTTAGT-3′
Caspase-3	F: 5′-GCAGCAGCCTCAAATTGTTGAC-3′ R: 5′-TGCTCCGGCTCAAACCATC-3′
Caspase-8	F: 5′-CCTGTTCTAAGCCTGTCTC-3′ R: 5′-TGGGAAGGAAGCCTCTAT-3′
Bax	F: 5′-GAGACACTCGCTCAGCTTCTTG-3′ R: 5′-TTGCTACAGGGTTTCATCCAGG-3′
Bcl-2	F: 5′-TGCAGATGCCGGTTCAGGTAC-3′ R: 5′-GGGAGCGTCAACAGGGAGATG-3′
GLAST	F: 5′-CATCTTGGTTTCGCTGTCT-3′ R: 5′-GGGGAACTCCGTGATTGA-3′
GLT-1	F: 5′-AAGCAGCCCGCCACATAC-3′ R: 5′-AACCGAGGGTGCCAACAA-3′
PAG	F: 5′-GCGTTCTCAGGCGGGATT-3′ R: 5′-TCAGCCATTCAGCGACCAG-3′
IL-1β	F: 5′-AGGAGAGACAAGCAACGACA-3′ R: 5′-CTTTTCCATCTTCTTCTTTGGTAT-3′
IL-6	F: 5′-ATGGGCCTTCTTGGGACTGATGT-3′ R: 5′-GGTCTGTTGTGGGTGGTATCCTC-3′

### Statistical analysis

Data analyses were performed using SPSS version 20.0 (IBM Corp., Armonk, NY, USA). All data are expressed as the mean ± SEM. In the case of the MWM, escape latency, swimming speed, and swimming distance were analysed using the two-way ANOVA of repeated measures, whereas one-way ANOVA was performed on the rest of the measured data analysis. Significance was defined at *P *< 0.05.

## Results

### Naringin improves the spatial learning and memory of CCL2-administered rats

**Fig. 2:**
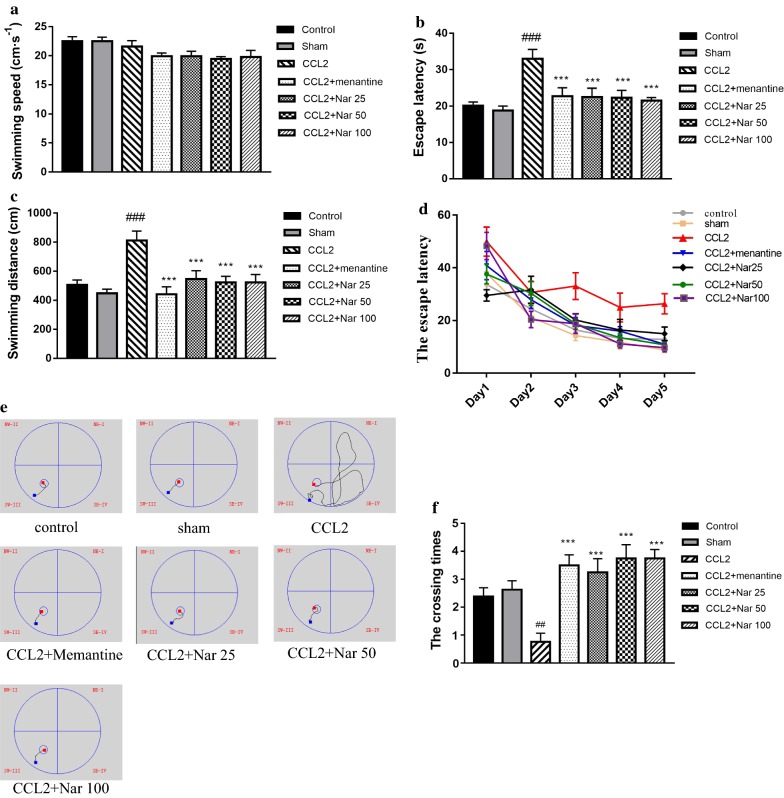
Naringin improves the learning and memory functions of CCL2 administered rats. Effect of Naringin on spatial learning in CCL2-treated rat were evaluated using the Morris water maze for five consecutive days. **a** The average swimming speeds among groups. There were no significance among groups. **b** The escape latency among groups. CCL2 group spent more time to found hidden platform compared with the Naringin treatment groups. **c** The average swimming distances among groups. The CCL2 treatment rats showed a significantly longer courses to arrived platform compaerd with the Naringin treatment groups. **d** The escape latency tendency during 5-day training. The time to found platform is shorten across days. **e** The typical track of searching for the hidden platform on training day 5. **f** The number of crossings of the target quadrant. The number of crossings of the target quadrant in the Naringin treatment groups significantly incresed compared with CCL2 group was observed. Values are expressed as mean ± SEM, control group, sham group, memantine group, Naringin low dose group, Naringin middle dose group, Naringin high dose group: *n *= 10;CCL2 group: *n *= 9; Positive drug group: n = 11 (^*#*^*P *< *0.05*, ^*##*^*P *< 0.01 vs sham, ^*^P < 0.05, ^**^P < 0.01 vs CCL2)

To evaluate the protective role of naringin in CCL2-induced spatial learning and memory impairment, we performed the MWM test. In spatial navigation training, the swimming speed for all groups demonstrated no significant differences (Fig. 2a). The escape latency of each group gradually decreased across the training days (Fig. 2d). Compared to the sham group, the escape latency of the CCL2 group was significantly increased; naringin treatment significantly decreased the escape latency (F_(6,63)_ = 5.448, *P *< 0.001, Fig. 2b). Additionally, the results of swimming distances were comparable to those of escape latency (F_(6,63)_ = 6.280, *P *< 0.001, Fig. 2c). The swimming path on training day 5 is shown in Fig. 2e. In the probe trial, the crossing times in the CCL2 group decreased significantly compared to the sham group; in the naringin-treated group, crossing times were significantly increased compared to the CCL2 group (F_(6, 63)_ = 4.794, *P *< 0.001, Fig. 2f).

### Naringin ameliorates recognition memory in CCL2-administered rats

**Fig. 3:**
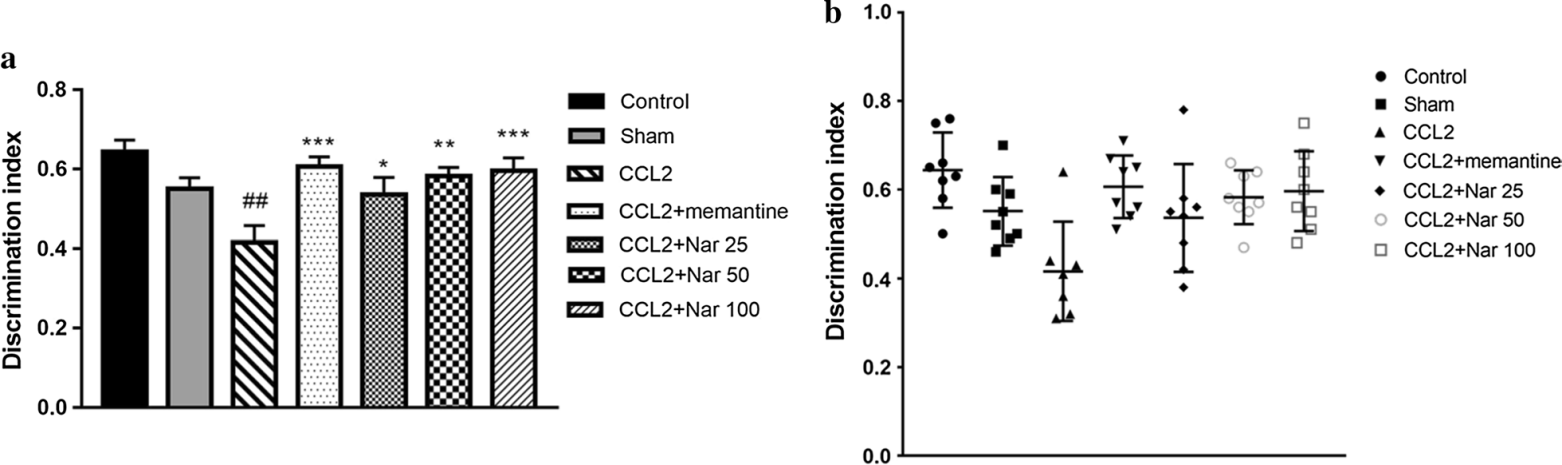
Naringin ameliorates recognition memory of CCL2–administered rats in novel object recognition. Effects of Naringin on cognitive function in CCL2-Induced rats was evaluate by novel object recognition test. **a** The discrimination index among group. CCL2 group rats showed significant lower discrimination index compared with the Naringin treatment groups. **b** The discrimination index of individual of each group. Naringin treatment groups rats spent more time to explore the novel object when compared to CCL2 group. Values are expressed as mean ± SEM, control group, sham group, Positive drug group, memantine group, Naringin low dose group, Naringin middle dose group, Naringin high dose group: *n *= 8; CCL2 group: *n *= 7 (^*#*^*P *< *0.05*, ^*##*^*P *< 0.01 vs sham, ^*^P < 0.05, ^**^P < 0.01 vs CCL2)

To further evaluate cognitive function, NORT was performed following the MWM assay. In the CCL2 group, the DIs was significantly reduced compared to the sham group. Compared to the CCL2 group, the naringin-treated groups demonstrated a significant increase in the DI in a dose-dependent manner (F_(6, 48)_ = 4.899, *P *< 0.001, Fig. 3).

### Naringin protects neurons in the hippocampal CA1 zone

**Fig. 4:**
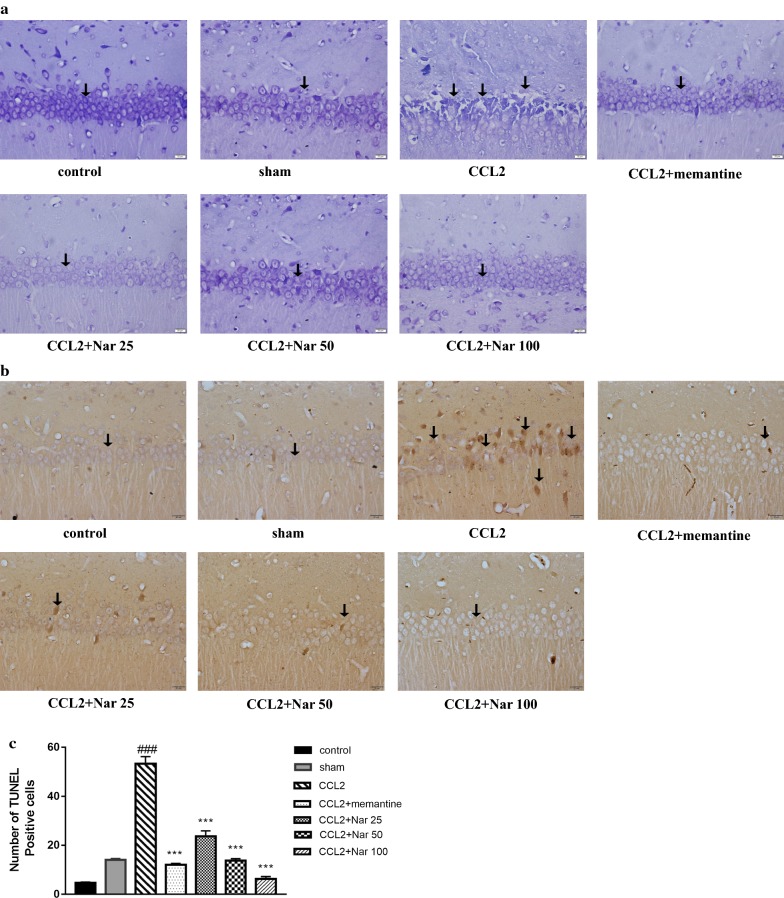
The effects of Naringin on neuroprotection. **a** Represents photomicrographs of Nissl staining of brain tissue sections across hippocampus CA1 region (magnification 400×). **b** Represents photomicrographs of TUNEL staining of brain tissue sections across hippocampus CA1 region,TUNEL staining to detect neuronal apoptosis in the brain tissues of rats, arrows indicate apoptotic cells (magnification 400×). **c** The TUNEL-positive cells. Values are expressed as mean ± SEM, n = 3 (^*#*^*P *< 0.05, ^*##*^*P *< 0.01 vs sham, ^*^P < 0.05, ^**^P < 0.01 vs CCL2)

Nissl staining revealed the morphological changes induced in the CA1 structure of the hippocampus. Compared to the sham group, the CCL2 group exhibited numerous damaged neurons in the hippocampal CA1 region, presenting indistinct cell boundaries, with small darkened and shrunken nuclei. In contrast, the naringin-treated groups demonstrated only minimal morphological changes (Fig. 4a). Furthermore, TUNEL staining was used to quantify the number of apoptotic neurons in the CA1 zone. In the CCL2 group, the number of the apoptotic neurons was significantly higher than that in the sham group; naringin treatment inhibited neuronal apoptosis (F_(6, 14)_ = 124.862, *P *< 0.001, Fig. 4b, c).

### Naringin reduces oxidative stress induced by CCL2 in rats

**Fig. 5:**
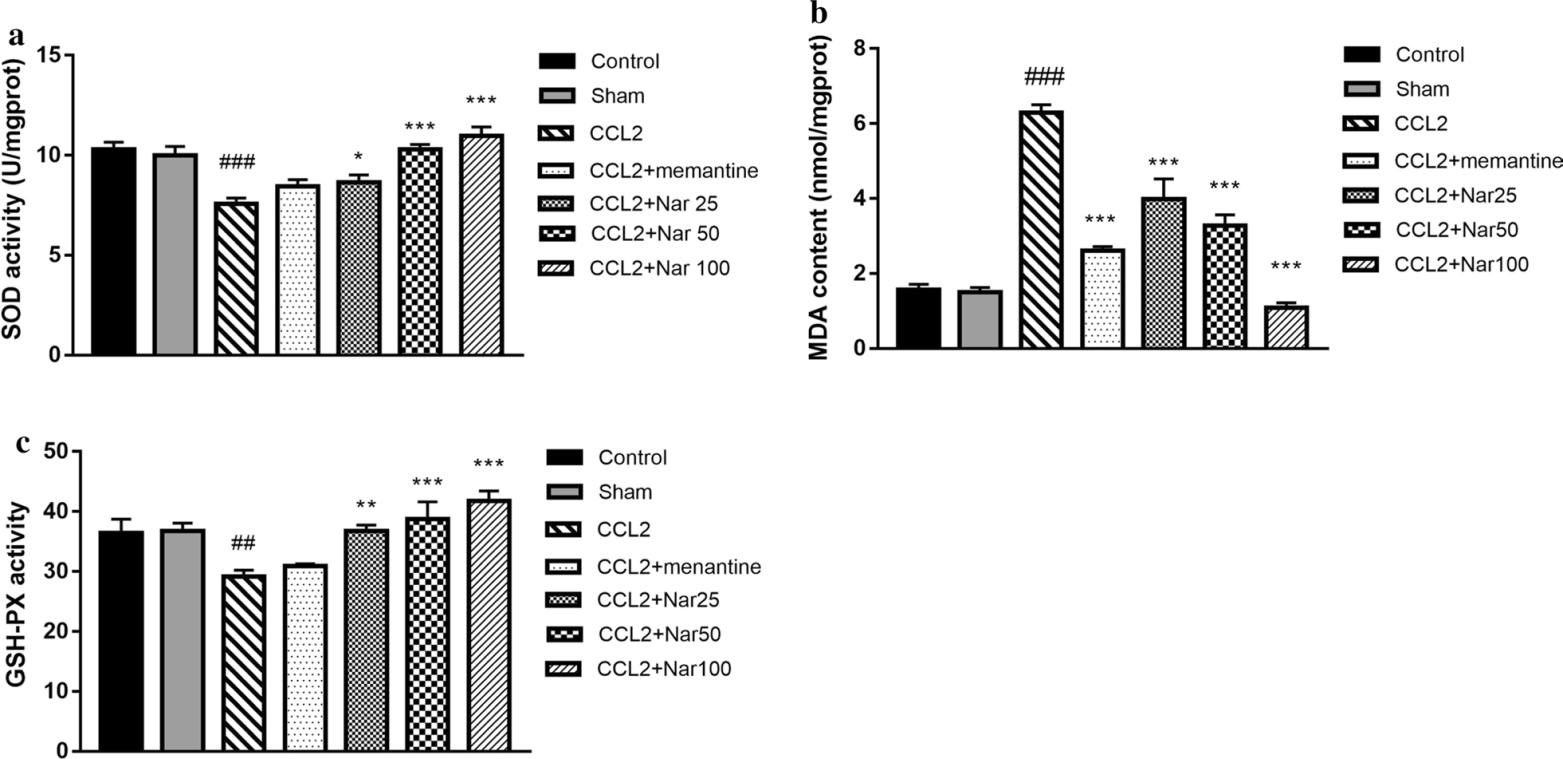
Effect of different dose of Naringin 25 mg/kg, Naringin 50 mg/kg, and Naringin 100 mg/kg on the oxidative stress status in CCL2 treated rats. The supernatant of hippocampus homogenate was used for the assay of SOD, GSH-PX activity and MDA levels. A significant increase in (**a**) SOD and (**c**) GSH-PX activity in Naringin treatment groups compared with model group. **b** A significant reduction in MDA levels in Naringin treatment groups compared with model group. Values are expressed as mean ± SEM, n = 8 (^*#*^*P *< 0.05, ^*##*^*P *< 0.01, ^*^P < 0.05, ^**^P < 0.01 vs CCL2)

In the hippocampus, the MDA content and enzymatic activity of SOD and GSH-Px were measured using commercial kits. Compared to the sham group, GSH‑Px and SOD activities were significantly decreased in the hippocampus of the CCL2 group, whereas the MDA content was significantly increased. Compared to the CCL2 group, naringin treatment significantly increased the activity of SOD (F_(6, 49)_ = 11.736, *P *< 0.001, Fig. 5a) and GSH‑Px (F_(6, 49)_ = 6.603, *P *< 0.001, Fig. 5c) while decreasing the MDA content (F_(6, 49)_ = 48.557, *P *< 0.001, Fig. 5b).

### Naringin decreases inflammatory-associated mRNA expression

**Fig. 6:**
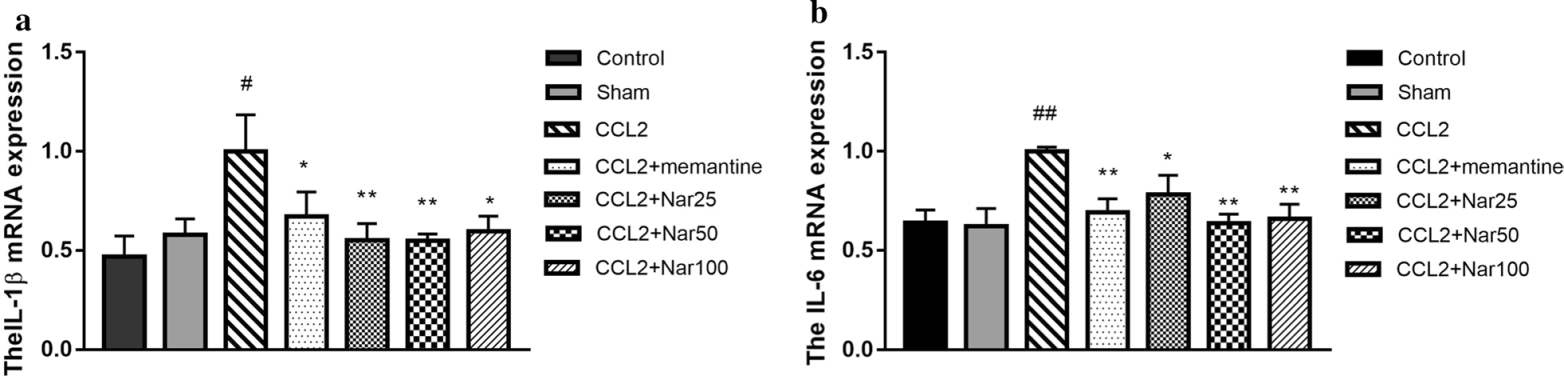
Effects of Naringin on IL-1β,IL-6 mRNA expression in the hippocampus of CCL2-treated rats. Quantitative real-time PCR analysis of messenger (m)RNA levels. Relative expression of: **a** IL-1βmRNA and **b** IL-6mRNA. qPCR analysis showed increased expression of IL-1β,IL-6 mRNA in Hippocampus of CCL2 rats. This result showing that Naringin significantly decreased expression of IL-1β, IL-6 mRNA in CCL2group rat.The experiments were repeated four times independently. Values are expressed as mean ± SEM, n = 4 (^*#*^*P *< 0.05, ^*##*^*P *< 0.01 vs sham, ^*^P < 0.05, ^**^P < 0.01 vs CCL2)

Here, we detected the mRNA expression of IL-6 and IL-1β to evaluate whether naringin could decrease the extent of CCL2-induced inflammation. In the CCL2 group, the mRNA expression levels of IL-6 and IL-1β were significantly higher than the sham group. Compared to the CCL2 group, naringin significantly decreased the mRNA expression of IL-6 (F_(6, 17)_ = 3.087, *P *< 0.05, Fig. 6a) and IL-1β (F_(6, 17)_ = 2.541, *P *< 0.05, Fig. 6b).

### Naringin regulates glutamate metabolism-associated mRNA expression

**Fig. 7:**
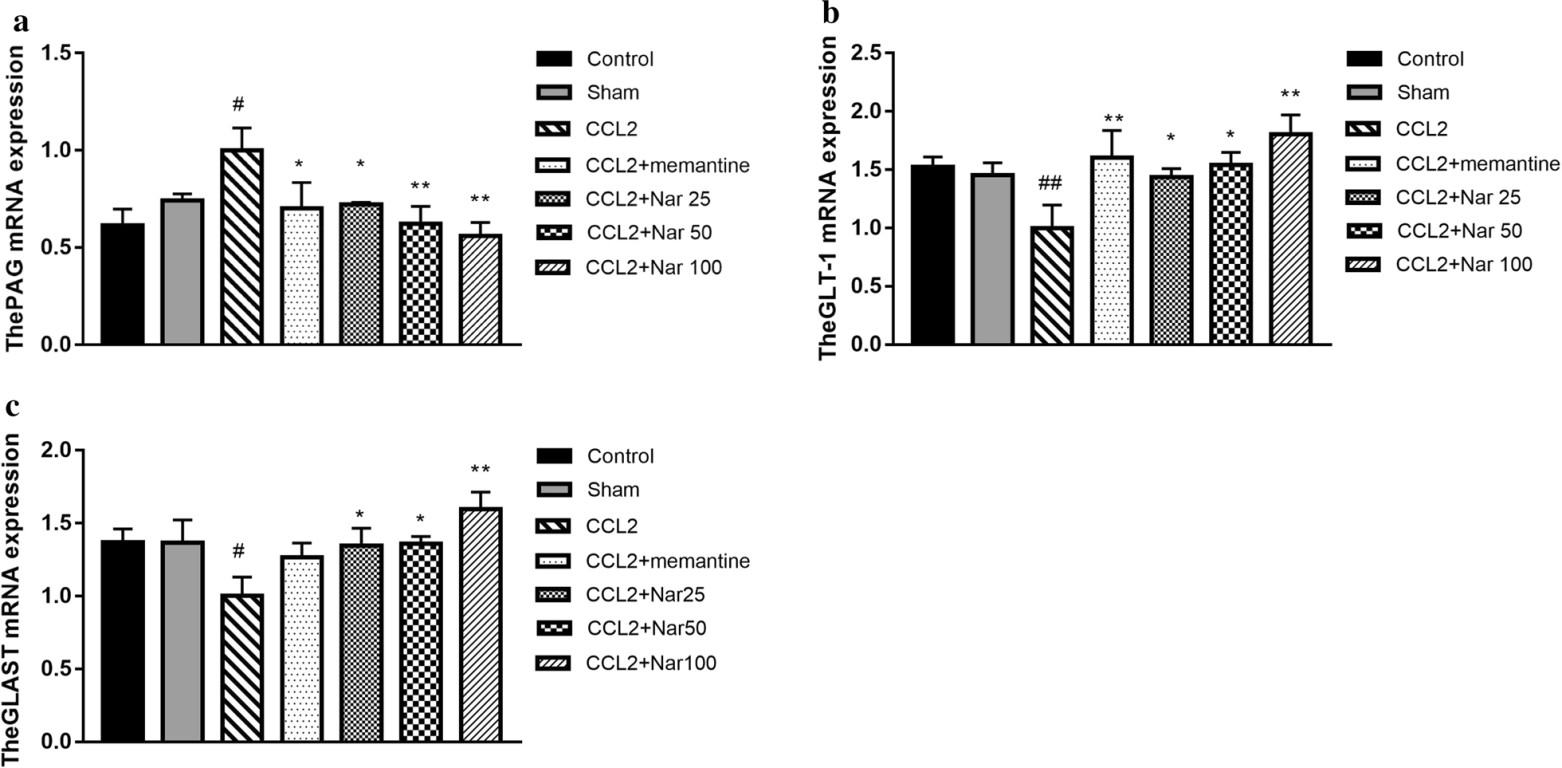
Effects of Naringin on PAG, GLAST, and GLT-1mRNA expression in the hippocampus of CCL2-treated rats. Quantitative real-time PCR analysis of messenger (m)RNA levels. Relative expression of: **a** The PAG mRNA. **b** The GLAST mRNA. **c** The GLT-1 mRNA. qPCR analysis showed increased expression of PAG mRNA while decreased expression of GLAST, GLT-1 mRNA in hippocampus of CCL2 rats, This result showing that Naringin significantly decreased expression of PAG mRNA while significantly increased GLAST, GLT-1 mRNA in CCL2 group rat.The experiments were repeated four times independently. Values are expressed as mean ± SEM, n = 4 (^*#*^*P *< 0.05, ^*##*^*P *< 0.01 vs sham, ^*^P < 0.05, ^**^P < 0.01 vs CCL2)

We detected the major regulators involved in glutamate metabolism, including glutamate transporter-1 (GLT-1), glutamate/aspartate transporter (GLAST), and phosphate-activated glutaminase (PAG). In the CCL2 group, the mRNA expression of PAG was higher than that in the sham group, whereas the GLAST and GLT-1 expression levels were lower. Compared to the CCL2 group, naringin significantly decreased the mRNA expression of PAG (F_(6, 17)_ = 2.582, *P *> 0.05, Fig. 7a) and increased the mRNA expression of GLAST (F_(6, 17)_ = 2.421, *P *> 0.05, Fig. 7b) and GLT-1(F_(6, 17)_ = 2.723, *P *< 0.05, Fig. 7c).

### Naringin decreases apoptosis-associated mRNA expression

**Fig. 8:**
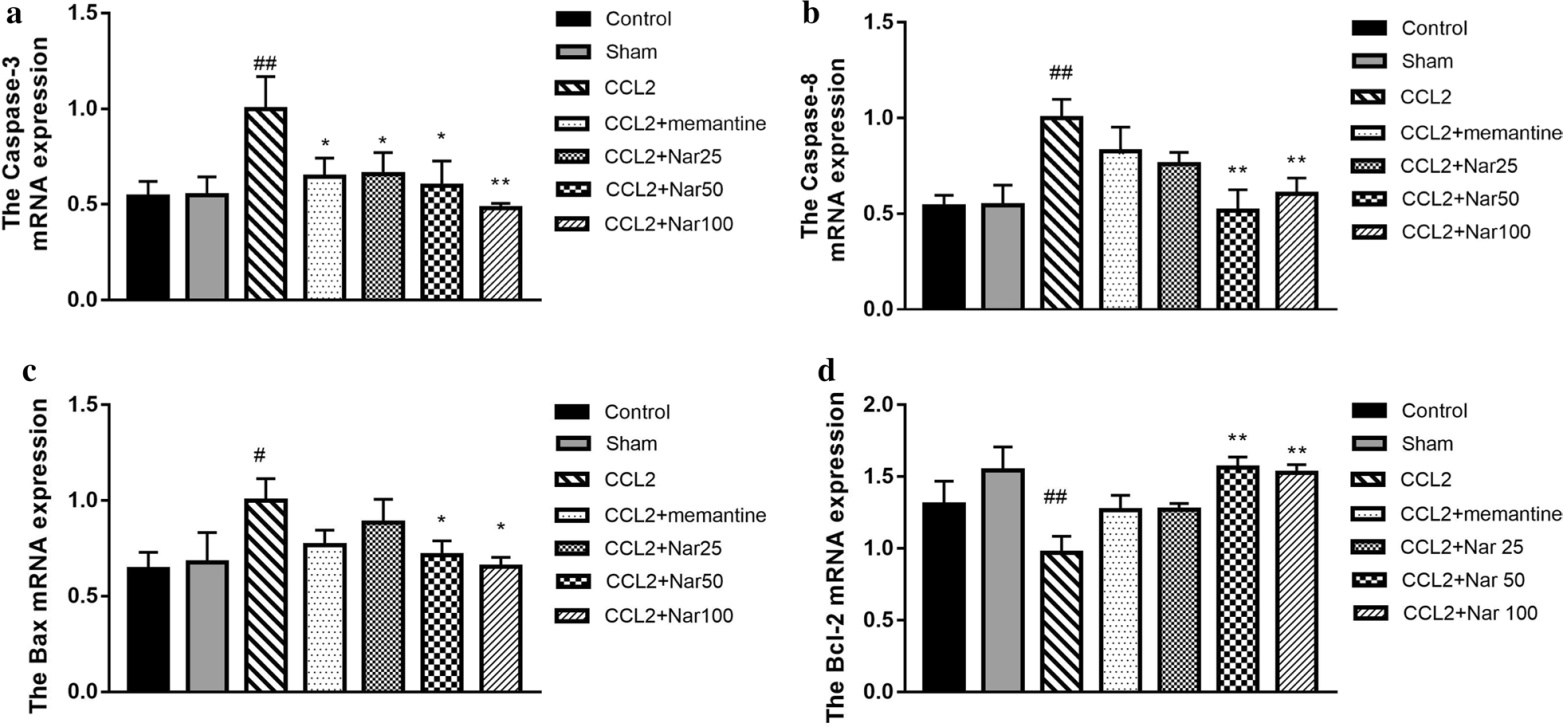
Effects of Naringin oncaspase-3, caspase-8,Bax,Bcl-2mRNA expression in the hippocampus of CCL2-treated rats. Quantitative real-time PCR analysis of messenger (m)RNA levels. Relative expression of: **a** The caspase-3 mRNA. **b** The caspase-8 mRNA. **c** The Bax mRNA expression. **d** The Bcl-2 mRNA. qPCR analysis showed increased expression of caspase-3, caspase-8, Bax mRNA while decreased expression of Bcl-2 in hippocampus of CCL2 rats, This result showing that Naringin significantly decreased expression of caspase-3, caspase-8, Bax mRNA while significantly increased Bcl-2 mRNA in CCL2 group rat.The experiments were repeated four times independently. Values are expressed as mean ± SEM, n = 4 (^*#*^*P *< 0.05, ^*##*^*P *< 0.01 vs sham, ^*^P < 0.05, ^**^P < 0.01 vs CCL2)

In the CCL2 group, the mRNA expression of cysteine aspartic acid-specific protease 3 (caspase-3), cysteine aspartic acid-specific protease 8 (caspase-8), and Bcl-2 associated X protein (Bax) were significantly upregulated, whereas cell lymphoma/leukaemia-2 (Bcl-2) was downregulated. Compared to the CCL2 group, naringin significantly decreased the mRNA expression levels of caspase-3 (F_(6, 17)_ = 2.269, *P *> 0.05, Fig. 8a), caspase-8 (F_(6, 17)_ = 3.545, *P *< 0.05, Fig. 8b), Bax (F_(6, 18)_ = 1.679, *P *> 0.05, Fig. 8c), and increased Bcl-2 mRNA expression (F_(6, 17)_ = 3.677, *P *< 0.05, Fig. 8d).

## Discussion

In addition to its well-characterised immune response function, CCL2 has recently demonstrated a pathophysiological role in several CNS diseases such as stroke, epilepsy, ischaemic brain injury [1], and neurodegenerative diseases [21, 22]. Previously, studies have reported increased CCL2 expression in the cerebrospinal fluid of patients with AD and HAND [6–9] in association with cognitive decline [6, 10]. Our previous study has demonstrated that CCL2 dose-dependently impairs spatial memory and object recognition in rats [23]. Furthermore, in-depth investigations have elucidated that the potential mechanisms are related to inflammation, oxidative stress, excitotoxicity, and neuronal apoptosis [6, 10]. Thus, CCL2, a multi-potent pathological factor, could be established as a therapeutic target to prevent or treat neurodegenerative diseases. However, currently available anti-CCL2 drugs remain limited. Thus, in the present study, we explored the neuroprotective effects of naringin against CCL2 induced damage.

The MWM and NORT are common behavioural paradigms used to evaluate spatial learning and memory and object cognition in rodents. In the MWM, swimming speeds did not significantly differ among groups, indicating that the surgery and drug treatment did not impair locomotor function in the experimental animals. In the CCL2 group, the escape latency, as well as the swimming distances, were significantly longer compared to the sham group; the crossing times were significantly decreased, confirming the detrimental effects of CCL2 in spatial learning and memory. Additionally, in the CCL2-treated group, NORT exhibited poor object recognition behaviour. However, naringin significantly improved cognition, as indicated by the shorter escape latency and swimming distances, increased crossing times, and DIs. Collectively, these results revealed the protective role of naringin against CCL2-induced cognitive impairment. Notably, a few studies have demonstrated that naringin improves learning and memory impairments induced by gp120, a crucial pathogenic factor of HAND pathogenesis [15].

In the brain, CCL2 is mainly produced by macrophages and microglia, the key mediators of neuroinflammation [24]. During inflammatory progression, CCL2 not only attracts immune cells to specific sites but also promotes the release of other inflammatory factors, including IL-6 and IL-1β, exacerbating the extent of neuroinflammation [10, 11]. Researchers have revealed that naringin demonstrates anti-inflammatory activity. For instance, naringin can attenuate increased tumor necrosis factor-α (TNF-α) levels in a kainic acid-induced animal model [25] and reduce periplaque-activated microglia and astrocytes in APP/PS1 transgenic mice [26]. Therefore, in this study, we examined the hippocampal expression of IL-1β and IL-6 mRNA to elucidate whether naringin could alleviate CCL2-induced neuroinflammation. In the CCL2 group, IL-1β and IL-6 mRNA expression significantly increased compared to the sham group; naringin treatment significantly decreased the mRNA expression of both interleukins, confirming the anti-inflammatory effects of naringin against CCL2-mediated neuroinflammation.

In general, neuroinflammation is accompanied by oxidative stress. Oxidative stress has been known to participate in the pathogenesis of several neurodegenerative diseases [27, 28]. A large number of reactive oxygen species (ROS) produced by oxidative stress can cause lipid peroxidation and DNA damage, provoking secondary neuronal damage, and ultimately damaging cognition [29]. Under normal circumstances, antioxidant defence systems, including antioxidative enzyme systems such as SOD and non-enzyme systems such as GSH-Px, could maintain equilibrium between the oxidative and antioxidative stress levels. For example, SOD scavenges free radicals and prevents lipid peroxidation in vivo to prevent oxidative damage. GSH-Px specifically catalyses hydrogen peroxide (H_2_O_2_) into water (H_2_O) to decrease the expression of H_2_O_2_ [16, 29]. Therefore, elevated SOD and GSH-Px activities could directly reflect a powerful antioxidative ability. In addition, the increased expression of MDA, as a major metabolite of lipid oxidation, reflects the oxidative degree [30]. Here, we observed that the expression levels of GSH‑Px and SOD were significantly reduced in the CCL2 group, whereas the expression of MDA was increased. This further confirmed the role of CCL2 in mediating oxidative stress. Naringin treatment markedly increased the expression of GSH‑Px and SOD in the hippocampus, and significantly reduced MDA levels. These results revealed the antioxidative stress effects of naringin. In fact, a few reports have revealed that naringin could ameliorate cognitive deficits by enhancing antioxidative stress [29, 31], as demonstrated by our current results.

In addition, our previous studies have observed that CCL2 can enhance NMDA receptor-mediated EPSC and mediate Ca^2+^ influx [12, 32]. This can impair the structure of neuronal dendrites in the hippocampal CA1 region and induce neuronal death, indicating that CCL2 can provoke excitotoxicity via a presynaptic mechanism and increase the release of glutamate [12, 32]. As the major excitatory neurotransmitter in the CNS, glutamate is involved in normal synaptic transmission and the process of long-term potentiation (LTP). However, the abnormal and excessive accumulation of glutamate in the synaptic cleft can trigger neuronal damage, termed as excitotoxicity. Physiologically, there is a glutamate-glutamine cycle between neurons and glial cells, mainly regulated by GLT-1, GLAST, and PAG. GLT-1 and GLAST are located in astrocytes; they take in the excessive glutamate and maintain normal neurotransmission. PAG is an enzyme located in the presynaptic terminal and catalyses glutamine to glutamate, which could enhance the level of glutamate [33, 34]. Therefore, the dysregulation of these regulators could lead to the excessive accumulation of glutamate in the synaptic cleft and eventually induce excitotoxicity [35–37]. Here, we tested the mRNA expression of PAG, GLAST, GLT-1 to elucidate whether naringin influenced glutamate metabolism. The results demonstrated that compared to the sham group, PAG mRNA expression levels increased, whereas the GLAST and GLT-1 mRNA expression levels decreased in the CCL2 group. In contrast, naringin treatment significantly reduced the PAG mRNA expression and increased the mRNA expression levels of GLAST and GLT-1, demonstrating that naringin has a protective effect on CCL2-induced excitotoxicity via the regulation of glutamate metabolism.

Apoptosis is a type of programmed cell death that clears the aging and necrotic organelles to maintain the normal physiological function in the body [38–40]. However, abnormal activation of apoptosis has been known to play a role in the pathophysiological processes of several diseases, including neurodegenerative diseases [12, 32]. In the CNS, neuroinflammation, oxidative stress, and excitotoxicity are the main factors inducing excessive neuronal apoptosis and cognition decline [29, 41–44]. The present results, combined with our previous research, demonstrated that CCL2 impaired cognitive function; the underlying mechanisms may associate with neuroinflammation, oxidative stress, and excitotoxicity. Thus, we proposed that the CL2 administration can induce hippocampal neuronal apoptosis. First, we observed the morphological changes in the hippocampal CA1 zones using Nissl and TUNEL staining. An obvious impairment of CA1 structure was observed in the CCL2 group via Nissl staining. Additionally, in the CCL2 treatment group, TUNEL staining demonstrated a significant increase in apoptotic hippocampal neurons in the CA1 zone, validating our hypothesis. Reportedly, naringin demonstrates anti-apoptotic effects in a cerebral infarction model [45] and quinolinic acid (QA)-induced neurotoxicity rat mode [46]. In our results, naringin treatment significantly protected the hippocampal neurons, consistent with the other observed outcomes. As apoptosis is regulated by a cascade of genes, we further examined the mRNA expression of caspase-8, caspase-3, Bax, and Bcl-2, to explore the apoptotic pathway. Caspase-8 is the upstream molecule that further activates caspase-3, a key apoptosis executive molecular [47, 48]. Furthermore, Bax and Bcl-2 are important mediators for apoptotic regulation. Bax is released from the mitochondrial inter-membrane space and amplifies the apoptotic signal. Conversely, Bcl-2 possesses anti-apoptotic effects [49–51]. In the model group, the mRNA expression levels of caspase-3,caspase-8, and Bax were upregulated, whereas the mRNA expression of Bcl-2 was downregulated. Naringin treatment significantly decreased the mRNA expression of caspase-3,caspase-8,and Bax, and significantly increased Bcl-2 expression, suggesting anti-apoptotic properties.

## Conclusion

**Fig. 9:**
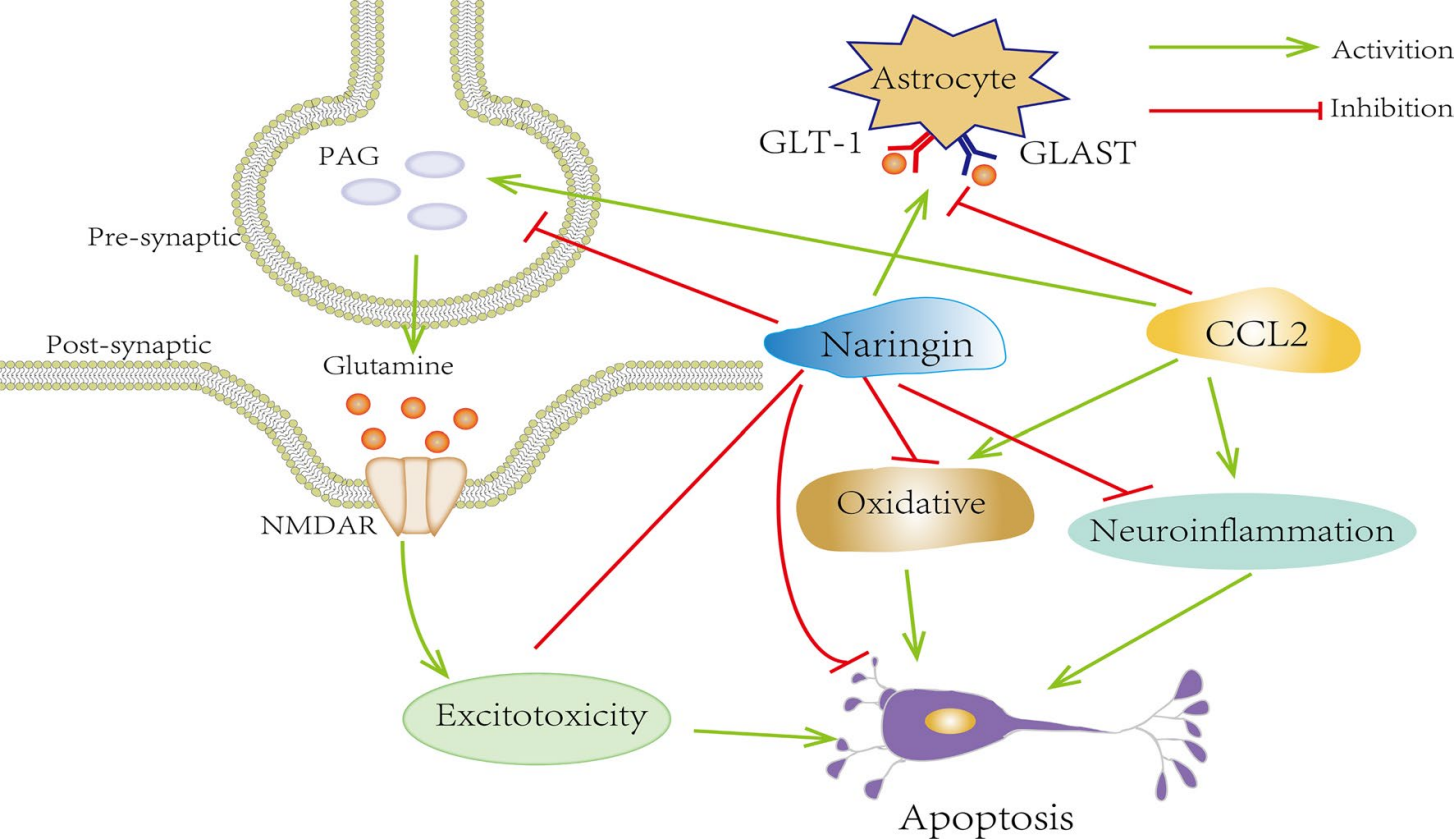
Naringin improves CCL2-induced cognition impairment mechanism illustration. Naringin treatment inhibited the oxidation, inflammation, reducing excitotoxicity and ultimately alleviating neurons apotosis and damage in rats with learning and memory impairment

In our study, we observed that naringin can afford protection against CCL2-induced cognition impairment; moreover, the underlying mechanisms were related to reduced inflammation, antioxidative stress, anti-apoptosis, and glutamate metabolism, indicating the potential neuronal protective effects of naringin as shown in Fig. 9.

## Data Availability

The datasets used and/or analysed during the current study are available from the corresponding author on request.
